# The Inflammasome in Times of COVID-19

**DOI:** 10.3389/fimmu.2020.583373

**Published:** 2020-10-08

**Authors:** Juan Carlos de Rivero Vaccari, W. Dalton Dietrich, Robert W. Keane, Juan Pablo de Rivero Vaccari

**Affiliations:** ^1^DRV Ventures, LLC, Miami, FL, United States; ^2^Department of Neurological Surgery and The Miami Project to Cure Paralysis, University of Miami Miller School of Medicine, Miami, FL, United States; ^3^Department of Physiology and Biophysics, University of Miami Miller School of Medicine, Miami, FL, United States; ^4^Center for Cognitive Neuroscience and Aging University of Miami Miller School of Medicine, Miami, FL, United States

**Keywords:** inflammasome, COVID-19, inflammation, coronavirus, caspase-1, IL-1beta

## Abstract

Coronaviruses (CoVs) are members of the genus Betacoronavirus and the Coronaviridiae family responsible for infections such as severe acute respiratory syndrome (SARS), Middle East respiratory syndrome (MERS), and more recently, coronavirus disease-2019 (COVID-19). CoV infections present mainly as respiratory infections that lead to acute respiratory distress syndrome (ARDS). However, CoVs, such as COVID-19, also present as a hyperactivation of the inflammatory response that results in increased production of inflammatory cytokines such as interleukin (IL)-1β and its downstream molecule IL-6. The inflammasome is a multiprotein complex involved in the activation of caspase-1 that leads to the activation of IL-1β in a variety of diseases and infections such as CoV infection and in different tissues such as lungs, brain, intestines and kidneys, all of which have been shown to be affected in COVID-19 patients. Here we review the literature regarding the mechanism of inflammasome activation by CoV infection, the role of the inflammasome in ARDS, ventilator-induced lung injury (VILI), and Disseminated Intravascular Coagulation (DIC) as well as the potential mechanism by which the inflammasome may contribute to the damaging effects of inflammation in the cardiac, renal, digestive, and nervous systems in COVID-19 patients.

Coronaviruses (CoVs) are members of the genus Betacoronavirus and the Coronaviridiae family. CoVs are enveloped positive-sense single stranded RNA viruses (~30 Kb in length) that encode for viral replicase. In addition, the outer envelope of CoVs is comprised of the spike (S), envelope (E), and membrane (M) glycoproteins; whereas the nucleocapsid (N) comprises the inner core (1). CoVs bind to angiotensin converting enzyme-2 (ACE2) receptors in host cells through the S protein (Figure 1) (2). The M and E glycoproteins are involved in viral assembly (3, 4) and the N protein coats the viral genome (5). In addition, CoVs also have the accessory proteins 3a, 3b, 6, 7b, 8a, 8b, and 9b.

**Figure 1:**
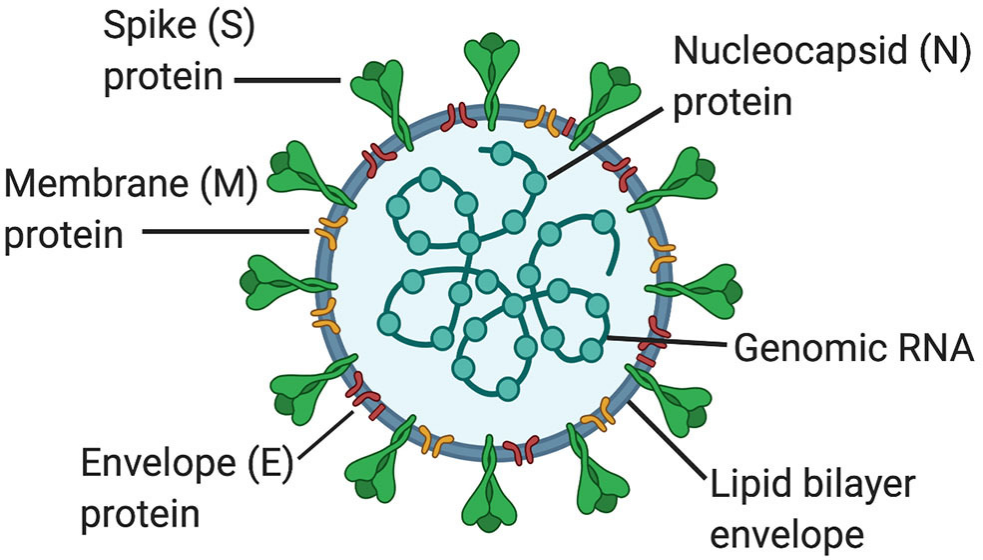
Structure of coronaviruses. In a lipid bilayer envelope, CoVs consist of a spike (S) protein, a membrane (M) protein and an envelope (E) protein. Inside the lipid bilayer envelope, the nucleocapsid (N) protein and the genomic RNA comprise the inner core.

In 2002, CoVs were responsible for severe acute respiratory syndrome (SARS) (6, 7); in 2012, CoVs were responsible for Middle East respiratory syndrome (MERS), and currently since 2019, the SARS coronavirus-2 (SARS-CoV-2) is responsible for coronavirus disease-2019 (COVID-19) (8).

It is evident that the rate at which new CoV infections are affecting humans is increasing. Thus, a better understanding of the mechanism of CoV infection is needed as well as the manifestations of symptoms and systemic complications associated with these infections in order to develop better therapies against COVID-19. Here we review the literature on the role of the inflammasome in CoV infections, which includes how CoVs activate inflammasomes upon infection, the role of the inflammasome in acute respiratory distress syndrome (ARDS), how ventilator-induced lung injury (VILI) activates the inflammasome, how the inflammasome plays a role in the systemic complications associated with COVID-19, and how the inflammasome is involved in the process of Disseminated Intravascular Coagulation (DIC).

## The Inflammasome

The inflammasome is a multiprotein complex of the innate immune response initially described as a regulator of caspase-1 activation and processing of the pro-inflammatory cytokines interleukin (IL)-1β and IL-18 (9). These multiprotein complexes are comprised of three basic components: (1) A sensor such as a NOD-like receptor (NLR) or an AIM-2 like receptor (ALR) (2) the adaptor protein apoptosis-associated speck-like protein containing a caspase-recruitment domain (ASC) and (3) the inflammatory cysteine aspartase caspase-1. Inflammasomes are named after their sensor proteins which include NLRP1, NLRP2, NLRP3, NLRC4, and AIM2, with NLRP3 being the most extensively studied inflammasome to date (10).

**Figure 2:**
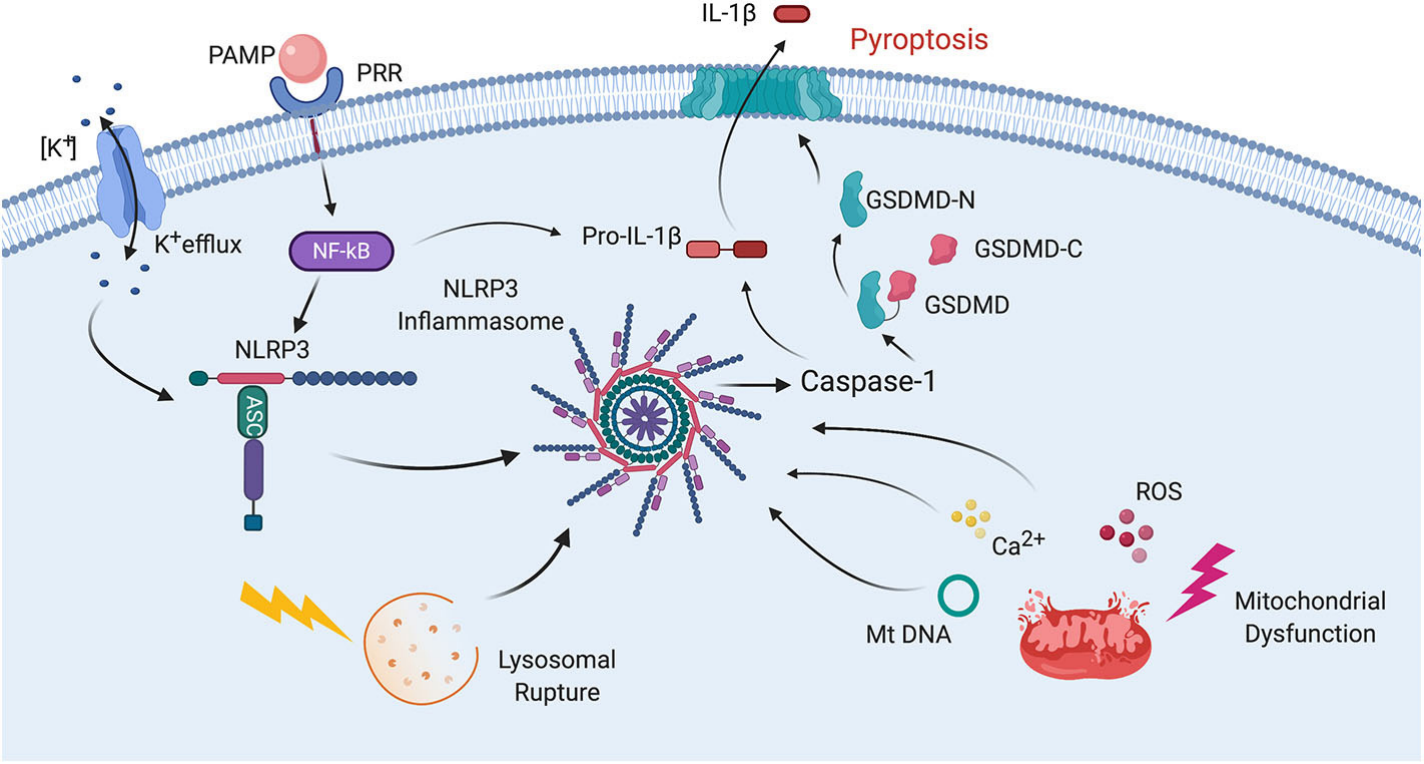
Mechanisms of inflammasome activation. Inflammasome activation in general relies on two signals for its activation. First, a PAMP binds to a PRR resulting in synthesis of NLRP3 and pro-IL-1β. Then a second signal leads to inflammasome assembly, leading to the activation of caspase-1, processing of pro-IL-1β into IL-1β and pyroptosis. The second signal may come from a variety of pathways including K^+^ efflux. Lysosomal rupture or mitochondrial dysfunction. Mitochondrial dysfunction results in release of ROS, Ca^2+^ and mitochondrial DNA (mtDNA), all of which have been shown to activate the inflammasome. Pyroptosis occurs as a result of caspase-1-mediated cleavage of GSDM-D at the linker region of GSDM-D. Following GSDM-D cleavage, the amino terminus of GSDM-D (GSDM-D-N) forms a non-selective pore at the cell membrane through which IL-1β is then released.

In addition to its role in cytokine production, the inflammasome is also involved in the cleavage of gasdermin-D (GSDM-D) in the cell death process of pyroptosis (11). GSDM-D is cleaved at the linker region between the amino (N) and carboxy (C) terminus by either caspase-1 and/or caspase-4 or -5 in humans (caspase-11 in rodents), resulting in freeing of the N-terminus from autoinhibiting the C-terminus, which allows formation of a pore by the N-terminus (GSDM-D-N) in the cell membrane (Figure 2) (12).

Inflammasomes were initially described for their role in mounting an innate immune response against bacterial (9), viral (13), and fungal (14) infections as well as in autoimmune diseases (15). However, lately, most attention has been paid to the role of inflammasomes in diseases such as rheumatoid arthritis (16), gout (17), diabetes (18), heart disease (19), renal diseases (20), hepatic diseases (21), psoriasis (22), vitiligo (23), multiple sclerosis (24, 25), Alzheimer's disease (26), Parkinson's disease (27), as well as central nervous system (CNS) injury (28–30), among others.

Each inflammasome is activated by different ligands which can either be endogenous or exogenous. Endogenous ligands are referred to as damage/danger-associated molecular patterns (DAMPs), and exogenous ligands are referred to as pathogen-associated molecular patterns (PAMPs). Examples of DAMPs include adenosine tri-phosphate or mitochondrial DNA (31). However, in the context of COVID-19, SARS-CoV-2 represents a PAMP capable of activating the inflammasome. During viral infections, inflammasomes play a role in the response to influenza virus (32), encephalomyocarditis virus (33), vesicular stomatitis virus (33), hepatitis C virus (34), respiratory syncytial virus (35), vesicular stomatitis virus (33), west Nile virus (36), Zika virus (37), swine fever virus (38), Ebola virus (39), H1N1 virus (40), Sendai virus (41), herpes simplex virus (42), Leishmania RNA virus (43), Dengue virus (44), human immunodeficiency virus (45), and CoVs (46).

The process of inflammasome activation involves a two-step process (Figure 2). The first step is referred to as signal 1 and represents the priming step of inflammasome activation in which a PAMP or DAMP binds to a pattern recognition receptor (PRR) such as toll-like receptor (TLR)-4 to stimulate the synthesis of pro-IL-1β and pro-IL-18 in a nuclear factor (NF)-κB-dependent manner (47). Once these pro-inflammatory cytokines are formed, then a second signal is needed to induce inflammasome formation and subsequent processing of pro-IL-1β and pro-IL-18 into its active forms which are then secreted by different mechanisms. One of these mechanisms includes the GSDM-D pore, which is formed by the N-terminus of GSDM-D that is inserted in the membrane (GSDMD-N) following GSDM-D cleavage (48). Inflammasome formation involves a process in which the sensor molecule such as NLRP3 oligomerizes and then the adaptor protein ASC is recruited into the complex, followed by incorporation of caspase-1, which is then autoproteolytically cleaved into its active form. This cleaved or active form of caspase-1 then exerts its catalytic activity on the pro-inflammatory cytokines that after their release perpetuate the inflammatory response (31). Although there is no unifying consensus regarding the mechanism of inflammasome activation, various processes have been proposed to contribute to the second signal of inflammasome activation such as high extracellular K^+^ concentration, K^+^ efflux, mitochondrial dysfunction, formation of reactive oxygen species (ROS), oxidized mitochondrial DNA, lysosomal degradation, and Ca^2+^ imbalance (Figure 2) (49, 50).

## The Inflammasome and Coronaviruses

**Figure 3:**
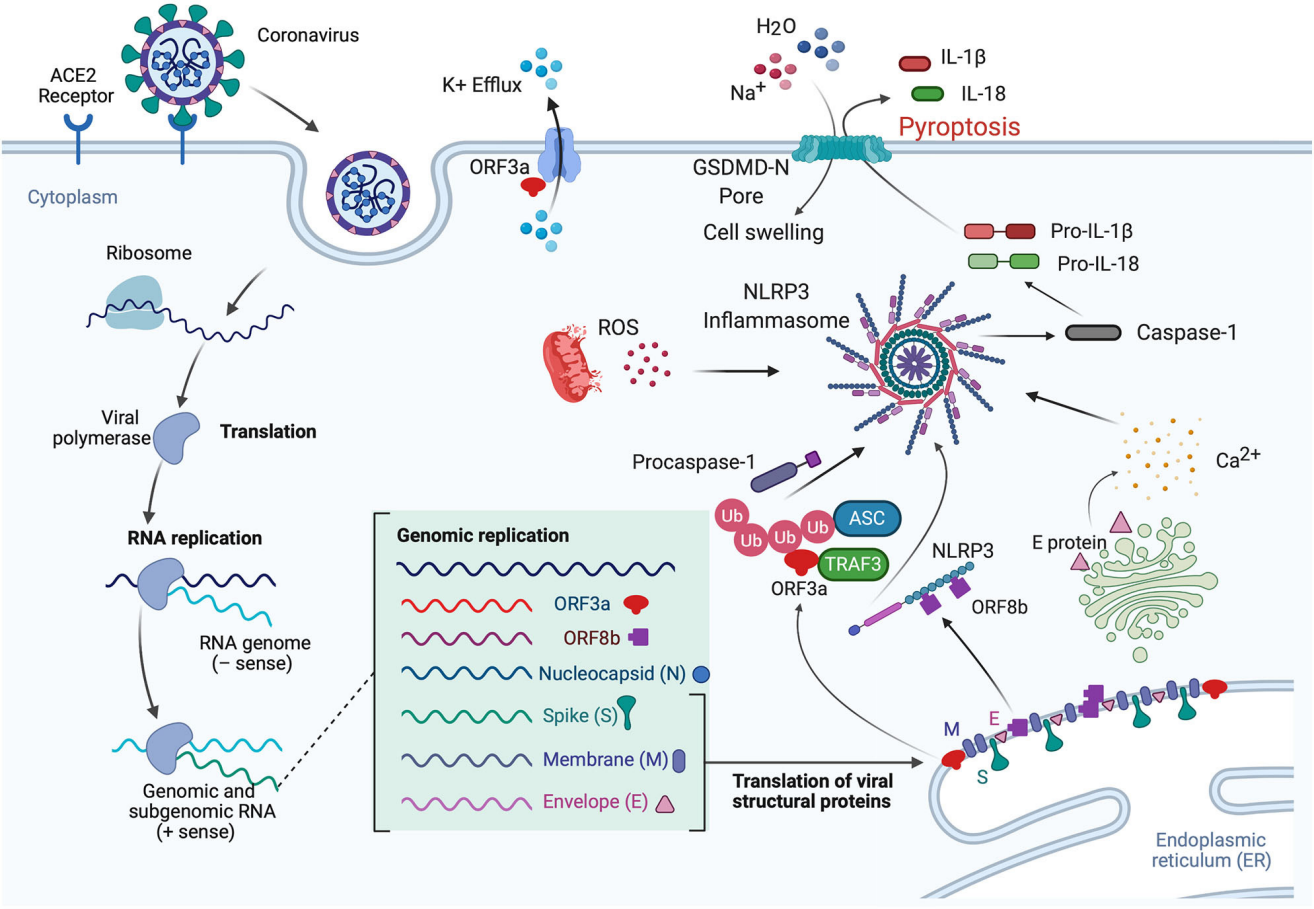
Mechanisms of inflammasome activation in CoV infection. In the lungs when the S protein of SARS-CoV-2 binds to the ACE2 receptor, the virus is internalized by endocytosis, leading to translation and RNA replication of genomic and sub-genomic RNA including ORF3a, ORF8b, and the viral structural proteins (N, S, M, and E proteins). The E protein is involved in Ca^2+^ release from the Golgi apparatus. This Ca^2+^ has the potential to activate the inflammasome. In addition, ORF3a interacts with TRAF3 to ubiquitinate ASC, and ORF8b interacts with NLRP3, resulting in inflammasome activation and pyroptosis. IL-1β is released through the GSDM-D-N pore during pyroptosis, while Na^+^ and H_2_O molecules enter the cell, resulting in cell swelling, which then manifests as pulmonary edema. Furthermore, ORF3a also acts in the cell membrane as a K^+^ channel, which causes an ionic imbalance also capable of promoting inflammasome activation, and mitochondrial dysfunction produces ROS that also contribute to inflammasome activation.

Viroporins are hydrophobic proteins that facilitate release of viral proteins from infected cells by modifying the cell membrane, and they are involved in the activation of inflammasomes. Viroporins are associated in viral pathogenesis, and their low ionic selectivity makes them ideal candidates for ionic exchange, which is critical for viral infection and inflammasome activation (Figure 3). When viroporins are blocked or deleted, the severity of infections tends to decrease (51), making viroporins attractive for the development of therapies to prevent exacerbation of the inflammatory response associated with viral infections (Table 1).

**Table 1:** CoV proteins and their role of inflammasome activation.

**Viroporin/ accessory proteins**	**Function**	**References**
E	• High levels of IL-1β detected in the lung parenchyma following CoV infection • Lack of ion exchange following SARS-CoV infection lowers IL-1β • Ca^2+^-mediated inflammasome activation	(52)
3a	• Acts as a K^+^ channel • NLRP3 inflammasome activation	(53)
ORF3a	• Na^+^ and Ca^2+^ channel • Promotes NLRP3 inflammasome activation by ubiquitinating ASC • Production of pro-IL-1β by NF-kB • Processes ASC-dependent/NLRP3-independent pro-IL-1β	(54)
ORF8b	• Binds to LRR in NLRP3 to induce inflammasome activation and IL-1β release • Causes macrophage pyroptosis • Forms insoluble protein aggregates that interact with ASC and NLRP3	(55)

The E glycoprotein of CoV forms a membrane pore that allows the passage of ions (56). Mice infected with E protein viruses developed pulmonary edema (46), which is characteristic of ARDS, and a main cause of death associated with CoV infections (57, 58). In addition to edema following pore activity mediated by the E protein, high levels of the inflammasome-mediated pro-inflammatory cytokine IL-1β have been detected in the lung parenchyma (52). This finding suggests the involvement of the inflammasome in the mechanism of CoV infection.

Lack of ion exchange through the E protein following SARS-CoV infection results in lower levels of IL-1β and lower immune-related pathology. The events associated with over-activation of the immune response tend to be more damaging to the host than the response associated with cell death in the host induced by the virus (59). Thus, a modulation of the immune inflammatory response is as critical, if not more, than preventing cell death induced by viruses. This observation makes the inflammasome, the master regulator of IL-1β, a key target for CoV infections. In addition to the role of viroporin E in Ca^2+^-mediated inflammasome activation, the accessory protein 3a, which potentially acts as a K^+^ channel, has also been shown to activate the NLRP3 inflammasome (Figure 3) (53).

Like viroporin E, SARS-CoV open reading frame3a (ORF3a) acts as an ion channel (Na^+^, K^+^, Ca^2+^) (60). However, in regards to inflammasome activation, it seems that ORF3a promotes NLRP3 inflammasome activation by modulating the ubiquitination of the inflammasome adaptor protein ASC and the production of pro-IL-1β by activation of NF-κB, which is independent of the ion-channel role that ORF3a plays. Furthermore, release of active IL-1β following activation of the NLRP3 inflammasome is dependent on tumor necrosis factor (TNF) receptor-associated factor 3 (TRAF3) (54). However, a similar effect was not detected for the AIM2 inflammasome. The effects of ORF3a on the priming (signal 1) and the processing (signal 2) of pro-IL-1β on inflammasome activation suggests a mechanism by which one protein is responsible for both signaling events needed for inflammasome activation, unlike other infectious mechanisms that rely on lipopolysaccharide (LPS) for the priming step (step 1) and adenosine tri-phosphate (ATP) for the activation step (step 2) (Figure 3). Interestingly, ORF3a is also able to process pro-IL-1β in an ASC-dependent but NLRP3-independent manner (54).

Shi et al. showed that ORF8b triggers NLRP3 inflammasome activation and IL-1β release by binding to the leucine rich repeat (LRR) domain of NLRP3, resulting in macrophage pyroptosis (55). In this study, the authors demonstrated that ORF8b formed insoluble protein aggregates. Moreover, ORF8b aggregates seemed to interact with NLRP3 and ASC forming a single speck (Figure 3). Considering that ASC specks have prionoid properties that interact with other pathogenic protein aggregates such a amyloid-β, resulting in a more exacerbated inflammatory response, a deeper understanding regarding the role of ORF8b aggregates following CoV infections would be beneficial to understanding the innate immune response mounted by these infections (61).

Consistent with previous studies regarding the mechanisms of inflammasome activation, it has been shown that Ca^2+^ imbalance is a common denominator in a variety of viral infections that result in inflammasome activation such as influenza (62), and encephalomyocarditis virus (63). Ionic imbalance has been associated with inflammasome activation in the lung following infections (64), and consistent with this finding is a recent study by Nieto-Torres et al. showing that E protein from SARS-CoV makes a Ca^2+^ permeable channel in the endoplasmic reticulum (ER)/Golgi intermediate compartment (ERGIC)/Golgi membrane that results in NLRP3 inflammasome activation and increased levels of IL-1β (Figure 3) (46).

The macrodomain of SARS Unique Domain (SUD) known as SUD-MC is involved in the activation of the chemokine CXCL10 and IL-1β in lung epithelial cells as determined by the levels of IL-1β in bronchioalveolar lavage fluid (BALF) (65). This process is mediated by the NLRP3 inflammasome in a c-Jun-dependent pathway. However, a similar effect was not detected in NLRP3 knockout mice, indicating that NLRP3 is the main inflammasome responsible for this SUD-MC-mediated effect.

In contrast, the inflammasome also plays a protective role following murine CoV infection (66). The mouse hepatitis virus (MHV) strain-A59 (MHV-A59) is a positive-strand RNA virus, like SARS-CoV-2, that is used to study CoV infections with effects in the CNS, liver, spleen, and lungs. Using this model, Zalinger et al. illustrated that inflammasome activation contributes to control of viral replication through IL-18. IL-18 knockout mice presented poor survival and increased viral replication (66). Furthermore, IL-18 was involved in production of interferon (IFN)-γ in activated T-cells. The authors showed that caspase-1 and caspase-11 knockout mice were more susceptible to MHV infection. Nonetheless, survival increased in IL-1 knockout mice when compared to wildtype, even when the viral load was higher in the IL-1 knockouts (66). Therefore, as a result of the different effects of each inflammasome signaling protein on viral load and survival, care must be taken when considering therapies that aim to block inflammasome activation for in certain infections, the lack of inflammasome activation may result in death, which is probably due to the negative consequences associated with a suppressed immune response (67). Accordingly, ASC and caspase-1 have been shown to be necessary for protective adaptive immunity against influenza. However, in that study, a similar role was not found for NLRP3 (32), yet other reports have shown an important involvement of NRLP3 following influenza infection (67, 68), which adds further complexity to the understanding of inflammasome signaling following viral infections.

MHV strain-3 (MHV-3) causes a viral fulminant hepatitis that results in production of fibrinogen-like protein-2 (FGL2), a monocyte/macrophage-specific procoagulant (69). This procoagulant effect may contribute to pathomechanisms that trigger DIC. Guo et al. showed that MHV-3 infection increased the levels of IL-1β in the serum and liver of mice. They also showed that the levels of FGL2 from macrophages was diminished in IL-1R1 knockout mice, and this finding was consistent with decreased infiltration of CD45^+^ Gr-1^high^ neutrophils. In addition, ROS derived from the NADPH oxidase complex (NOX) resulted in NLRP3 inflammasome activation; thus, NLRP3 and caspase-1 knockouts showed lower levels of IL-1β (70).

## Coronaviruses and Inflammation

The inflammatory response following CoV infection has an anti-viral and a pro-viral role. In regard to the anti-viral response, inflammation restricts viral replication and infection. However, inflammation plays a pro-viral role when it acts to release virions. Current understanding of SARS-CoV-2 infection indicates that the virus infects the cell through angiotensin-converting enzyme-2 (ACE2) receptors in host cells by binding to the S glycoprotein. ACE2 is part of the renin-angiotensin system (RAS) and is involved in the regulation of blood pressure and fluid homeostasis (71).

Recently, Shao et al. demonstrated that ACE2 receptor stimulation results in NLRP3 inflammasome activation in podocytes; thus, leading to cell death. Interestingly, this effect did not affect blood pressure (72). However, whether SARS-CoV-2 binding through the S glycoprotein to ACE2 results in inflammasome activation has yet to be determined. In addition, binding of angiotensin II receptor 1 (AT1) also results in NLRP3 inflammasome activation (73).

Interestingly, bats that are known to be infected by several CoVs such as Ebola, MERS, SARS-Co-V, and potentially SARS-CoV-2 remain asymptomatic following infection even in the presence of high viral loads in blood and tissues (74). It has been suggested that COVID-19 was passed to humans by an intermediate host between bats and humans, similar to previous CoV infections that were transmitted to humans through camels (MERS) or civets (SARS-CoV). Nevertheless, the intermediate host for COVID-19 remains unknown. Recently, Ahn et al. showed that following infection with MERS-CoV, bats are able to fight CoV infections with lower levels of NLRP3 inflammasome activation when compared to humans, without affecting viral load (75). Therefore, the molecular mechanism employed by bats to limit the damaging effects of CoV infections should be explored to develop better interventions for the care of COVID-19-positive patients.

## Inflammasomes, Acute Respiratory Distress Syndrome, and Pneumonia

Traditionally, acute lung injury (ALI) is defined by pulmonary infiltrates and edema present in the chest as determined by radiography procedures in the absence of left atrial hypertension, or a pulmonary wedge pressure lower than 18 mmHg and an arterial oxygen to inspired oxygen fraction (PaO_2_/FiO_2_) lower than 300 mmHg (76). On the other hand, when the PaO_2_/FiO_2_ is below 200 mmHg the ALI is referred to as ARDS (76). Hence, according to this definition ARDS is a more severe form of ALI. However, the modern definition of ARDS, eliminates the use of ALI for humans, and limits its use to animal studies. Moreover, this modern definition of ARDS divides ARDS into mild (300–200 mmHg), moderate (200–100 mmHg) and severe (below 100 mmHg) based on the Berlin definition (77).

More recently, ARDS has been stratified based on different phenotypes such as those that are physiologically derived, which separates patients according to the PaO_2_/FiO_2_ ratio, the pulmonary dead space, the ventilator ratio, and the driving pressure (78, 79). The clinically derived phenotype considers whether the etiology is direct (pulmonary origin) or indirect (extrapulmonary origin) (78, 79). The biologic phenotype relies on biomarkers associated with ARDS and considers whether there is a hypo or hyperinflammatory response, which can be used as a guide for potential therapies (78, 79). Examples of these inflammatory markers that are associated with the cytokine storm are IL-6, IL-8, IL-18, and TNF. In addition, other markers associated with ARDS are proteins of endothelial injury such as surfactant protein-D or coagulation-associated proteins such as plasminogen activator inhibitor-1 and protein C. Moreover, recently it has been shown that increased levels of IL-18 were consistent with increased mortality in sepsis-induced ARDS (80). Therefore, the hyperinflammatory phenotype is characterized by increased inflammation, less ventilator-free days, and increased mortality when compared to the hypoinflammatory phenotype (81, 82). Thus, supporting a strong role for the inflammatory response in ARDS that is capable of determining favorable or unfavorable outcomes depending on whether there is hyperinflammation or hypoinflammation. Finally, another phenotype that has been described is the omics derived phenotype which stratifies patients based on genome-wide association and microRNA transcriptomic analysis (78, 79).

Of the well-known CoVs (HCoV-OC43, HCoV-NL63, HCoV-HKU1, MERS-CoV, SARS-CoV, and SARS-CoV-2), SARS-CoV-2 has garnered especial attention due to the level of infectivity as well as lethality in vulnerable populations. The acute stage of CoV infections is characterized by infiltration of immune cells into lung tissue, whereas the post-acute stage is characterized by pulmonary fibrosis (83). COVID-19, like other CoV infections, causes ALI with high viral titers, high levels of the inflammatory cytokines IL-1β and IL-6 as well as infiltration of macrophages and neutrophils into the lungs (84).

High mobility group box 1 protein (HMGB1), which activates the inflammasome in the lungs leading to ARDS/ALI (85), is upstream of IL-6 release (86), and has been suggested to play a key role in the inflammatory response occurring in the lungs of COVID-19 patients (87).

COVID-19 infections are associated with bacterial and viral pneumonia. Pneumonia following CoV infection can be either viral that may result in secondary bacterial pneumonia, or due to a combination of viral and bacterial pneumonia, However, the combined type has a lower incidence. Following SARS-CoV infection, secondary bacterial (methicillin-resistant *Staphylococcus aureus*) pneumonia has been described with ventilator-associated pneumonia (VAP) (88).

Although the role of the inflammasome in viral pneumonias has not been thoroughly examined, several studies point to inflammasome activation after bacterial infections by different organisms. For instance, NLRP3, ASC, and caspase-1 are upregulated following *Streptococcus pneumoniae (S. pneumoniae)* infection, resulting in production of IL-1β (89). The authors showed that NLRP3 knockout cells were able to produce IL-1β. However, ASC knockouts significantly decreased the levels of IL-1β (89), pointing to the possibility that even if the NLRP3 inflammasome is blocked, other inflammasomes that require ASC may compensate for the role of NLRP3 such as the AIM2 inflammasome or other NLR-dependent inflammasomes, yet NLRP3 knockout mice are more susceptible to the effects of pneumococcal pneumonia infection than wild types. Moreover, levels of infiltrated leukocytes into the lungs was not affected by knockdown of NLRP3, but pulmonary edema did increase in the NLRP3 knockout mice as determined by decreased dynamic lung compliance, which probably explains why NLRP3 knockouts were more likely to die. However, a better measure of edema would have been the determination of the wet to dry lung ratio or BALF total protein.

Similarly, mice deficient in NLRP3 are also susceptible to the effects of α-hemolysin-expressing *Staphylococcus aureus* (*S. aureus*) in murine pneumonia and are able to produce IL-1β, suggesting that other inflammasomes besides NLRP3 may be involved in the innate immune response to S. aureus pneumonia (90). On the other hand, knocking out the NLRC4 inflammasome has been shown to be protective following *Pseudomonas aeruginosa* (*P. Aeruginosa*) pneumonia as determined by improved bacterial clearance, decreased mortality and decreased lung damage (91). In this study, the authors suggested that the inflammasome may not be needed to fight the infection. However, the inflammasome seemed to play a role in increasing levels of IL-1β and IL-18, resulting in decreased bacterial clearance and increased lung toxicity. In contrast, during influenza A infection, the AIM2 inflammasome is activated, leading to lung injury and mortality (92). Moreover, in this study the authors showed that AIM2 knockout mice presented less ALI and increased survival without affecting viral load in the lungs (92).

## COVID-19, Ventilator Support, and the Inflammasome

Mechanical ventilation is used as a treatment for ARDS in order to expand collapsed alveoli. The high pressure generated by mechanical ventilation leads to VILI (93). VILI has been described in SARS (94) and COVID-19 (93). Wu et al. showed that this process was mediated in part by the NLRP3 inflammasome by sensing lung alveolar stretch (95), suggesting that stretch-injury in VILI activates an innate immune response that was partially mediated by the inflammasome. In addition, activation of the NLRP3 inflammasome by stretched injury seems to be regulated by an interaction between NEK-7 and NLRP3, which can be treated with glibenclamide (Glyburide) in mice (96).

Furthermore, a study by Dolinay and colleagues showed upregulation of *Il1b* in a rodent model of VILI (97). The mRNA transcript levels of caspase-1, IL-1β, and IL18 were higher in patients with sepsis/ARDS when compared to patients with systemic inflammatory response syndrome and controls (97). Moreover, deletion of IL-18 and caspase-1 were shown to be protective following VILI, and delivery of a neutralizing antibody against IL-18 resulted in decreased neutrophil counts in BALF (97).

Moreover, another complication associated with mechanical ventilation in COVID-19 patients is VAP (98, 99). In general, VAP takes place in ~20% of patients who undergo mechanical ventilation for over 48 h (100), resulting in ~30% mortality rate (101). *Escherichia coli* (*E. coli*), *Klebsiella pneumoniae, P. aeruginosa, Acinetobacter baumannii*, and *S. aureus* are the main causative organisms of VAP (102). Mortality in the intensive care unit associated with VAP is usually related to multi-drug resistant pathogens, and early diagnosis of VAP by proper identification of the causative agent is paramount to increase patient survival (98). In a study analyzing BALF and serum from 73 patients suspected to have VAP and 21 age-matched volunteer controls, it was found that IL-1β and IL-8 in BALF were higher in the VAP suspected cases when compared to controls (103).

## Inflammasomes, cytokine Release Syndrome, And COVID-19

It has been suggested that a major contributor to poor outcomes in patients with COVID-19 is an exacerbated immune response (104). This heightened immune response is characterized by unusually high levels of inflammatory cytokines such as IL-1β (the main cytokine activated by the inflammasome together with IL-18), IL-2, Monocyte Chemoattractant Protein-1 (MCP-1), macrophage inflammatory protein-1α (MIP1A), IL-6, IL-7, IL-8, TNF, Granulocyte-macrophage colony-stimulating factor (GM-CSF), CC-chemokine ligand 2 (CCL2), CCL3, CXC-chemokine ligand 10 (CXCL10), and the soluble form of the α-chain of the IL-2 receptor, among others (104, 105). The exacerbated immune response is referred to as cytokine storm syndrome or cytokine release syndrome (CRS). However, despite the increased levels of a variety of cytokines in COVID-19 patients, those protein levels seem to be 10 to 40 times lower in COVID-19 patients than in patients with ARDS (106). Thus, CRS may not fully explain the poor outcomes experienced by COVID-19 patients and further investigation into the inflammatory response in these patients is granted.

The heightened inflammatory response in COVID-19 patients presents with decreased CD-8^+^ T cells in blood (lymphopenia) probably due to infiltration of these cells into tissues or due to a response to the steroid treatment given to these patients (104). In post-mortem studies, lymphocytic cell death has been detected in the lymph nodes and spleen, which could also explain the lymphopenia. CRS may result in hemophagocytic lymphohistiocytosis (HLH) or macrophage activation syndrome (MAS), leading to high fever, high levels of ferritin, and hypertriglyceridemia (107).

Symptoms of CRS range from mild to high fever, fatigue, headache, rash, arthralgia, myalgia, hypotension, circulatory shock, vascular leakage, DIC, and multi-organ dysfunction syndrome (MODS) (107). Patients with CRS also present with cytopenia, and elevated C-reactive protein (CRP), creatinine levels, liver enzymes, and D-Dimer values. Additionally, von Willebrand factor (VWF), a marker of endothelial activation is also increased in CRS and has been described in COVID-19 patients (108). This suggests that endothelial cells may be an attractive therapeutic target for the treatment of COVID-19-related hyperinflammation, especially in cases presenting capillary leakage, hypotension and coagulopathy.

IL-6 is induced by IL-1β, the main cytokine activated by the inflammasome (109). Therefore, inhibition of the inflammasome could be expected to help treat patients with CRS. IL-18, the other cytokine controlled by inflammasome activation is also elevated in patients with CRS (110). Since there is no FDA-approved drug that directly inhibits the inflammasome, to this extent, anti-IL-6 and anti-IL-1β signaling therapies are being tested in patients with COVID-19 (111, 112). Increased IL-6 levels lead to vascular leakage, DIC and myocardial dysfunction (107).

In addition, type I IFN signaling has been reported to be decreased in patients with COVID-19 (113). It is possible that decreased type I IFN signaling in the presence of an exacerbated inflammatory response may be related to increased inflammasome signaling (114). However, viral infections are capable of generating high levels of type I IFN, and deletion of IFNAR1, a receptor involved in type I IFN signaling, or downstream type I IFN signaling pathways increases the replication, dissemination and lethality associated with viral infections (115), indicating that type I IFN are also involved in viral clearance.

In a mouse model of *S*. *suis* infection, Lin et al. studied the systemic effects of Streptococcal toxic-shock-like syndrome (STSLS) on cytokine production. STSLS is characterized by fever, blood spots (purpura), hypotension, shock and MODS, similar to what is seen in patients with CRS. In that study, the NLRP3 inflammasome was activated by *S. suis* leading to production of IL-1β, resulting in CRS (116), further highlighting that the inflammasome is a contributor to the effects of CRS following infections. Thus, the cytokine storm results in MODS, which can severely affect patients by inducing an inflammatory response that spreads to other organs beyond the lungs. As a result, a concern in COVID-19 patients is not only what happens due to the pulmonary infection but also the non-respiratory manifestations associated with the inflammatory response caused by SARS-CoV-2 infections.

## The Inflammasome, COVID-19, and Non-Respiratory Manifestations

In addition to the respiratory effects associated with COVID-19, other manifestations affecting a variety of organ-systems have been recognized (Table 2).

**Table 2:** Potential effects of inflammasome activation on COVID-19 manifestations.

**Manifestation**	**Effects**	**References**
Respiratory	• HMGB1 activates the inflammasome leading to ARDS and ALI • HMGB1 is upstream of IL-6 NLRP3, ASC and caspase-1 are upregulated following *(S. pneumoniae)* infection	(85, 86, 88, 89)
Cardiovascular	• Pressure overload, activates the inflammasome in the heart following stimulation of b-adrenergic receptors, resulting in higher levels of NLRP3, ASC and IL-18 from myocardial cells • Cardiac arrhythmias • IL-1β and IL-18 have been associated with hypertension	(117–121)
Gastrointestinal	• Enteroviruses activate the inflammasome by acting on NLRP3, caspase-1, ASC, IL-1β and GSDM-D • Ionic imbalances associated with SARS-CoV-2 infections may activate the inflammasome • NLRP3 inflammasome inhibition lowers ALT and AST levels, improving liver fibrosis • Acute pancreatitis activates the NLRP3 inflammasome • The gut-lung axis communicates the lungs with the GI system	(122–125)
Neurological	• The brain-lung axis communicates the lungs with the brain through EV containing inflammasome proteins	(126)
Ophthalmic	• In conjunctival goblet cells, infections activate the NLRP3 inflammasome • In conjunctival goblet cells, infections activate the purinergic receptors P2X4 and P2X7 • The NLRC4 inflammasome is activated in corneal ulcers	(127, 128)
Renal	• Zika virus induces AKI through NLRP3 inflammasome	(129)

### Cardiovascular Manifestations

Patients with pre-existent cardiovascular conditions tend to have worse outcomes due to COVID-19 than patients who do not present a cardiovascular comorbidity, including hypertension. Due to the manifestation of a cardiovascular involvement in more severe cases, it is likely that the effects on the heart are due to sequelae associated with the CRS. Thus, a reduction and control of the cytokine storm may alleviate the deleterious effects in these patients. COVID-19 exacerbates underlying cardiovascular conditions such as ischemic heart disease and chronic heart failure (130). In addition, it may cause myocardial injury, myocarditis, arrhythmia, acute coronary syndrome, cardiogenic shock, stroke, venous thromboembolism, and pulmonary embolism (130).

Non-ischemic events in the heart, such as pressure overload, activate the inflammasome in the heart probably as a result of ROS production following stimulation of β-adrenergic receptors, resulting in higher levels of NLRP3 and ASC as well as production of IL-18 from myocardial cells (117). Similarly the inflammasome was shown to be involved in cardiac arrhythmias (118), and higher levels of IL-1β and IL-18 have been associated with hypertension (119). In a mouse model of hypertension, the inflammasome is activated in the kidneys, resulting in production of IL-1β but not IL-18 (120); whereas inhibition of the inflammasome resulted in lower blood pressure (121).

Taken together, considering the exacerbated inflammatory response that COVID-19 patients present, the effects of the inflammasome on the cardiovascular system could explain, in part, some of the adverse cardiovascular events seen with COVID-19; however, a direct role between inflammasomes and cardiac complications has not been tested in an animal model of CoV infections.

### Gastrointestinal Manifestations

Problems with the gastrointestinal system such as diarrhea, abdominal pain, vomiting and lack of appetite have been described in COVID-19 patients. In some cases, these symptoms occur even in the absence of any respiratory symptoms, and sometimes correspond to a longer time between disease onset and hospitalization when compared to patients who do not present any digestive symptoms (131). Lack of appetite may be associated with the anosmia that characterizes some of the symptoms experienced by some patients. However, the cause of digestive symptoms in some COVID-19 cases is not well-understood, yet it is possible that the effects are due to an alteration of the intestinal microbiome by the infection, or the result of the effects of the virus binding to the liver, which expresses ACE2 receptors. Accordingly, Pan et al. suggest that an alteration in the gut-lung axis (122) may be responsible for the digestive symptoms in some COVID-19 patients, which could also explain how a problem affecting the lungs also affects the gastrointestinal system (131).

Similar to CoVs, enteroviruses are also positive-sense single stranded RNA viruses, and several enteroviruses have been shown to activate the inflammasome by acting on NLRP3, caspase-1, ASC, IL-1β, and GSDM-D (123), indicating that the inflammasome can be activated by infections that affect the gastrointestinal system, and that the machinery responsible for the inflammatory response mediated by the inflammasome is present in the gastrointestinal system. However, whether CoVs activate the inflammasome directly in the gastrointestinal tract is yet to be determined. It is possible that the ionic imbalances associated with SARS-CoV-2 infections also results in inflammasome activation in the gut (132), Moreover, there is ample evidence on inflammasome regulation of the inflammatory response in intestines in chronic diseases like Chron's disease and colitis (133), further highlighting the relevance of this innate immune complex in inflammatory events in the gastrointestinal tract.

In the liver, COVID-19 increases the levels of alanine aminotransferase (ALT) and aspartate aminotransferase (AST), particularly in severe cases (134). Although no-link between inflammasome and liver problems have been described following CoV infections, previous studies have shown that inhibition of the NLRP3 inflammasome lowers the levels of ALT and AST, improving outcomes in liver fibrosis and non-alcoholic steatohepatitis (NASH) in mice (124), suggesting that inflammasome inhibition may be beneficial to control the effects caused by CoVs infections in the liver.

The pancreas also expresses ACE2 receptors, making the pancreas a target for CoV infections. Previously, SARS-CoV was shown to damage pancreatic islet cells leading to diabetes (135). Furthermore, following COVID-19, there is an association between poorer outcomes and diabetes. However, poorer outcomes in diabetics seemed to be less frequent in older individuals and those with hypertension (136). However, the mechanism for this finding is presently unknown. It is possible that medications used to treat these patients also might serve to treat some of the symptoms associated with COVID-19, including hyperactivation of the immune response.

In addition, NLRP3 inflammasome proteins are elevated in monocyte-derived macrophages and peripheral blood mononuclear cells from patients with diabetes (137). Acute pancreatitis, which has been described in COVD-19 patients and is worse in diabetics, also activates the NLRP3 inflammasome (125). Thus, a heightened inflammatory response in patients with diabetes could be a risk factor in patients with diabetes and COVID-19.

### Neurological Manifestations

Cerebrovascular complications, convulsions, encephalitis, change in mental status, confusion, headaches and febrile seizures as well as taste (hypogeusia/ageusia) and smell (anosmia/hyposmia) dysfunction have been described in patients with COVID-19 (138, 139). Although, the mechanism of taste and smell dysfunction is not known, it is possible that the virus binds to ACE2 in the oral mucosa (140), affecting this sensing function. Although smell and taste dysfunction are common in several upper respiratory infections, it seems that in COVID-19, these are even more prevalent (141).

Moreover, CoVs have been detected in the cerebrospinal fluid (CSF) of patients with SARS-CoV (142), suggesting the possibility of a similar neurological involvement in patients with COVID-19. In addition, SARS-CoV-2 has been shown to affect human neural progenitor cells and brain organoids (143), indicating the possibility of direct infection by CoV in brain tissue. Inflammasomes have been shown to be activated in a variety of diseases and injuries affecting the CNS (25, 28–30). Thus, the CNS is capable of mounting an immune response through the inflammasome. Recently a mechanism was described by which inflammasome proteins are carried in extracellular vesicles (EV) to the lungs from the brain following brain injury, thus inducing ALI (126). Therefore, it is also possible that the lung secretes inflammasome proteins in extracellular vesicles that are carried to the CNS following infection, causing neurological symptoms. Although EV are capable of carrying viral components (144), whether EV are secreted during COVID-19 as part of the neuro-respiratory lung axis is yet to be determined.

### Ophthalmic Manifestations

Studies indicate that patients with severe COVID-19 may present conjunctivitis (145), and CoVs have been previously detected in tears (146). CoVs gain direct access to the conjunctival mucosa as the viral particles from an infected patient travel in droplets that reach the eye. However, how CoV reach the tear film in the absence of direct access from the virus to the conjunctival mucosa is yet to be fully elucidated. Evidence from feline CoV suggests that infected macrophages and monocytes extravasate immune tissues and cause endothelial cell dysfunction that leads to vasculitis (147). It is the vasculitis that is believed to be an underlying contributor to the ocular manifestations seen following feline CoV infections, which include conjunctivitis, retinal vasculitis, pyogranulomatous anterior uveitis and choroiditis with retinal detachment (148). In addition, in mouse CoV, inflammation in the eye results in optic neuritis and retinal degeneration that affects photoreceptors and ganglion cells (149).

Although conjunctivitis remains the only ocular manifestation widely reported in regards to COVID-19, it is possible that other conditions may arise upon closer examination of COVID-19 patients.

Moreover, in goblet cells of the conjunctiva S. aureus activates the NLRP3 inflammasome as well as the purinergic receptors P2X4 and P2X7 which have been shown to be involved in inflammasome signaling (49, 127, 150). In addition, *S. pneumoniae* and *P. aeruginosa* activate the NLRC4 inflammasome in corneal ulcers (128). Thus, the ocular surface has the inflammasome machinery necessary to mount an innate immune response against conjunctivitis in the presence of COVID-19. However, further studies are needed to understand the type of conjunctivitis present in COVID-19 patients.

### Renal Manifestations

Acute kidney injury (AKI) is a significant problem in patients with COVID-19 (151). Patients develop AKI during hospital admission and when disease is severe, such as in patients with ARDS or on mechanical ventilation, as well as in patients with hypertension or diabetes (151). It is possible that in COVID-19, the exacerbated inflammatory response or vascular thrombosis can damage the kidneys. In addition, ACE2 receptors are present in the kidney, making this organ a potential direct target of SARS-CoV-2 infection. A potential contributor to the exacerbated inflammatory response is the inflammasome that besides being involved in CRS, it is present in the kidneys where it contributes to inflammation in several renal diseases and complications, including AKI (152). Previously, the zika virus has been shown to induce AKI through NLRP3 inflammasome activation (129); however, whether SARS-CoV-2 is responsible for inducing AKI through the inflammasome in COVID-19 has yet to be tested.

## Inflammasome and Disseminated Intravascular Coagulation (DIC)

Severe cases of COVID-19 may present with coagulation complications that manifest as thrombi, high levels of D-dimers (a sign of fibrin degradation), prolonged prothrombin time, and low platelet count (thrombocytopenia) that may lead to DIC. Thrombi in COVID-19 patients have been described in the lungs, heart, brain, liver, kidneys and lower limbs (104). DIC tends to present in cases of sepsis where it blocks microvessels and leads to organ dysfunction.

**Figure 4:**
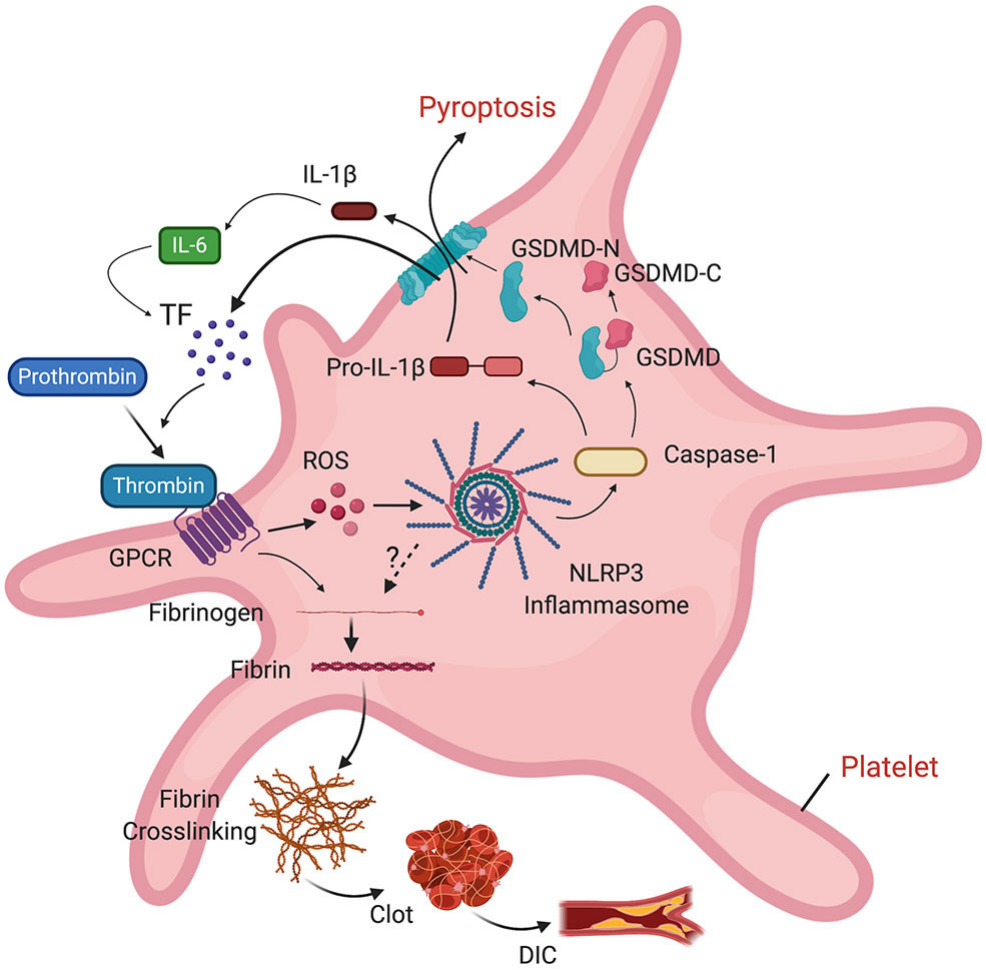
Role of the inflammasome on clot formation. Thrombin binds to a GPCR in platelets, resulting in ROS-dependent activation of the inflammasome and release of IL-1β into the cell. IL-1β stimulates production of IL-6. IL-6 stimulates tissue factor (TF) to convert prothrombin into thrombin. TF-containing microvesicles are released by pyroptosis following inflammasome activation. Thrombin then converts fibrinogen into fibrin, leading to fibrin cross-linking, clot formation and DIC.

In addition to DIC, some patients with COVID-19 may present pulmonary embolism (153) and deep vein thrombosis (154). The NLRP3 inflammasome has been described as a signaling intermediate between inflammation and thrombosis by modulating clot retraction and platelet spreading (155). NLRP3 knockout mice present abnormal hemostasis and arterial thrombosis, probably as part of a mechanism that involves binding of thrombin to G protein-coupled receptors (GPCR), which stimulates ROS production in platelets. ROS activates the inflammasome, resulting in IL-1β signaling that is followed by platelet spreading and clot retraction (155). IL-6 stimulates tissue factor (TF) to transform prothrombin into thrombin. Thrombin then converts fibrinogen into the fibrin that is characteristic of thrombi. Thus, there is a clear association between the inflammasome and clot formation. Furthermore, inflammasome activation causes release of microvesicles containing TF by pyroptosis, resulting in systemic coagulation and death (156), which provides a mechanism by which DIC may contribute to the poor outcomes experienced by COVID-19 patients (Figure 4).

## Conclusions

To date, therapies intended to treat COVID-19 include Remdesivir or Favipiravir to control translational replication of viral RNA, Tocilizumab to block the IL-6 receptor, Bevacizumab to block vascular endothelial growth factor (VEGF), Anakinra to block IL-1 receptor activity, Lopinavir or Ritonavir to target proteolysis, Losartan to target ACE2 receptors, corticosteroids such as Dexamethasone to target the exacerbated inflammatory response, heparin to treat DIC and intravenous immunoglobulins to target CRS, or convalescent plasma, among others (157). Thus, great efforts are being undertaken to develop therapeutics against the pulmonary and systemic manifestations of COVID-19.

This review highlights the inflammasome as a target to interfere with different aspects associated with this pandemic-causing virus. However, care must be taken since inflammasome signaling may be necessary to fight the actual viral infection, while at the same time inflammasome activation may be responsible for the hyperactivated inflammatory response that leads to sepsis, DIC, AKI and death. Mechanisms employed by bats to dampen CoV infections indicate that inflammation signaling pathways are probably better targets than reduction of viral load in controlling COVID-19. Thus, a better understanding of the role of inflammasomes and inflammatory processes in CoVs and those regulating viral load are critical for development of therapeutics to treat these diseases, and although to date there are no FDA-approved drugs that directly target the inflammasome, in regards to inflammasome signaling and therapeutics that can be considered for COVID-19 treatment, potential therapies that are currently being manufactured for the treatment of inflammasome-related diseases include MCC950 that interferes with NLRP3 inflammasome activation by binding to the NACHT domain of NRLP3, hence preventing its oligomerization (158), as well as IC100, a monoclonal antibody with intracellular and extracellular action that interferes with ASC speck formation (25) (Table 3).

**Table 3:** Potential therapies targeting the inflammasome that could be used in the care of COVID-19 patients.

**Therapy**	**Mechanism of action**	**References**
Dexamethasone	Decreases airway inflammation by inhibiting NLRP3 inflammasome activation and levels of IL-1β and IL-18	(159, 160)
Enoxaparin	Low molecular weight heparin shown to inhibit inflammasome activation in a mouse model of brain injury-induced ALI.	(161, 162)
IFN-β	Type I IFNs decreased NLRP3 inflammasome activation through STAT1	(163, 164)
MCC950	Inhibits inflammasome activation by preventing NLRP3 oligomerization	(158)
IC100	Inhibits inflammasome activation by preventing ASC-speck formation	(25)
M5049	Inhibits TLR7 and TLR8, which have been described in Inflammasome activation	(165–167)
Anakinra	IL-1 receptor blocker	(168)
Tocilizumab	Therapeutic monoclonal antibody that blocks IL-6 signaling	(168)

On the other hand, there are some therapies that are already FDA-approved and have been shown to interfere with inflammasome signaling activation which are already being considered for the treatment of COVID-19 such as Enoxaparin (161, 162), anakinra (168), tocilizumab (168), and dexamethasone (159, 160). Moreover, another drug that is already approved by the FDA that can also be used to inhibit the inflammasome is IFN-β (163), which is already used to treat multiple sclerosis (169), and is currently being tested for its effects on COVID-19 patients (164).

Moreover, TLR7 and TLR8, which have been implicated in inflammasome signaling (165) have been suggested to play an underlying role in COVID-19 severity (166). TLR7/8 are activated by single stranded RNA viruses like SARS-CoV-2, and in addition to their role on inflammasome activation (165), these PRR are better known for their involvement in type I IFN synthesis and a variety of IFN stimulated genes (ISG), which when deregulated are capable of contributing to an exacerbated inflammatory immune response (167). As a result, M5049, a TLR7/8 inhibitor, is currently being tested in clinical trials for the treatment of severe symptoms of COVID-19 as a potential treatment for CRS (167) (Table 3).

Given the number of people that have been affected with COVID-19 worldwide, a better understanding of the systemic effects associated with COVID-19 during and after infection resolution is of paramount importance, and given the possibility that CoV infections may become a seasonal infection similar to influenza virus infections, it remains critical to identify FDA-approved and future novel therapeutics that can be used for the treatment of CoV infections and the associated systemic manifestations.

## Author Contributions

JPdRV and JCdRV designed the structure of the review and performed the literature search. All authors contributed to the writing of this article.

## Conflict of Interest

JCdRV is employed by DRV Ventures, LLC. JPdRV, RWK, and WDD are co-founders and managing members of InflamaCORE, LLC and have licensed patents on inflammasome proteins as biomarkers of injury and disease as well as on targeting inflammasome proteins for therapeutic purposes. JPdRV, RWK, and WDD are Scientific Advisory Board Members of ZyVersa Therapeutics. JCdRV has licensed a patent on inflammasome proteins as biomarkers.
